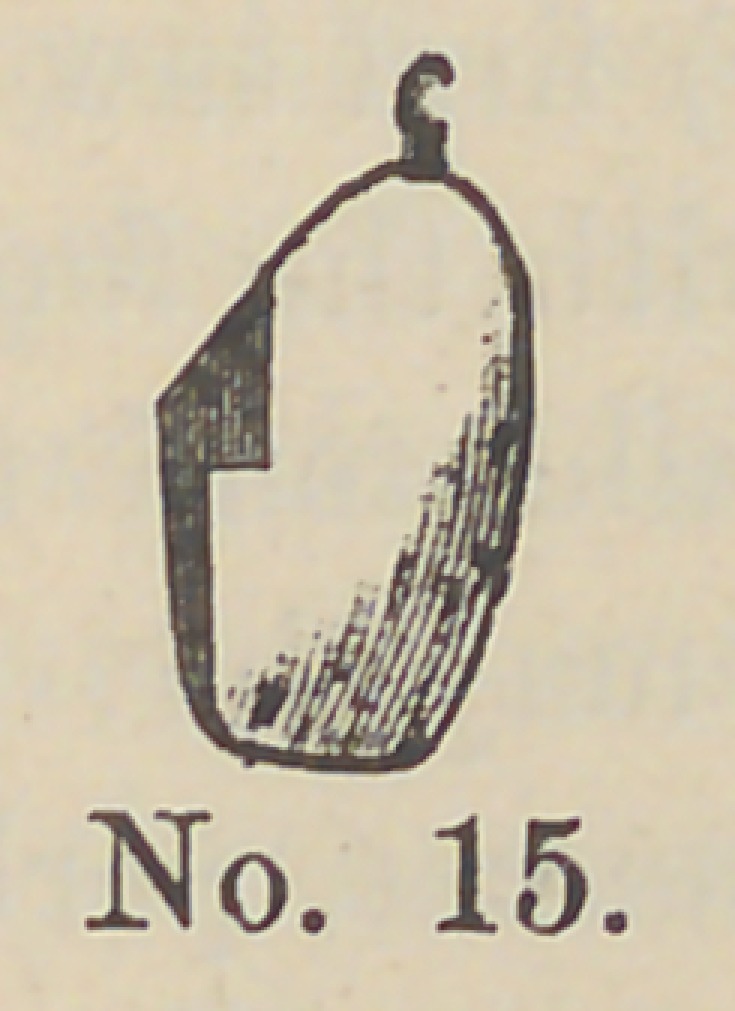# Bridge Work

**Published:** 1886-05

**Authors:** James E. Low


### Bridge Work.

(Read before the Mississippi Valley Dental Society.)

BY JAMES E. LOW.

The question, how can the partial loss of teeth be restored with the least possible injury and inconvenience to the wearer, is of so much importance that there is but one question greater to the conscientious dentist and patient, that of preserving the natural teeth; thereby avoiding the necessity of interfering with the natural condition. For thousands of years the importance of these organs, not only to masticate our food properly, but to keep in harmony and preserve a symmetrical appearance of the combined features of the face has been recognized. In the early history of mankind the bones of animals were carved in the shape of teeth and worn. Holes were drilled on either side of the denture and tied to the teeth adjoining with ligatures. These crude dentures with all their imperfection were considered ornamental to persons without teeth, although of but little use, if any, for mastication.

Notwithstanding their crudeness, and the fact that the acids of the mouth penetrated the bones so carved and made them offensive, the balance was drawn in their favor, and the higher classes wore them. This seems to have been almost the first manner of restoring the partial loss of teeth. Where roots remained, transplanting was practiced among a certain class able to pay some poor person for losing theirs.

This in most cases proved unsatisfactory, like nearly all previous attempts to restore partial loss of teeth, and I might add, nearly all of the present. Sometime later, natural teeth, those lost by disease of the gums and those of dead people, were used to supply vacancies, by first making a plate to fit the vacancy to be supplied. Clasps were soldered to this narrow plate, which rested on the gum to hold the denture in position by clasping the teeth adjoining those to be supplied. Pins were soldered to these narrow plates for each tooth to be supplied; holes were drilled longitudinally into the pulp cavity in the teeth; the teeth were then slipped over the pins and riveted fast on the lingual side.

Thus the toothless sufferers struggled along from the use of white wax crowded between the teeth and blocks carved from bones attached with strings and wires twisted around the natural teeth with screws inserted into the natural root to hold them in position and natural teeth fastened to clasps as stated, until the porcelain tooth was invented.

This obviated one of the principal objections, it being incorruptible, and not affected by the acids of the mouth. But the objectionable clasp attachments still remained, causing much trouble and annoyance by their constant motion on the teeth clasped, which in many instances soon became sensitive, and often loosened.

The next supposed advance was to abandon the clasp attachment, and cover the roof of the mouth, holding the denture in place by atmospheric pressure. The clasps were supposed to be more objectionable than the discomfort and inconvenience of wearing the plate over the roof of the mouth; but time and experience have thoroughly proven that more harm has resulted from their use than the clasp plate, as they not only injure the natural teeth by their constant motion, but most plates of this class are fitted close around the necks of the teeth, and cause recession of the gums by the inflammation produced; not only this, but thousands of persons wearing these plates covering the roof of the mouth were soon found to be suffering from inflammation and thickening of the mucus membrane, red and inflamed spots appearing there, and often, where there was a lagging of vitality, either from blood poison or otherwise, these plates set up inflammatory action, which many times caused the loss of the palate, vomer and nasal bones. Then again, in order to digest certain kinds of food, in fact all of the carbo-hydrates, such as starch, sugar, oil, gelatine, gum and flesh, the combined secretions of the follicles and salivary glands are needed and these could not be had where the roof of the mouth was covered. Consequently if we are not able to digest these as nature designed, the injury liable to occur physically and mentally, is incalculable.

Now, by a careful analysis of the fluids of the mouth, the salvary gland excretion will be found alkaline, and that of the mucus membrance, acid. Who doubts that these numerous follicle glands were for an all-wise purpose? We cannot close our eyes against these facts if we have any regard for humanity at large. We all know that inflamed mucus membrane does not excrete normal secretion, and that nine-tenths of all persons wearing the roof of the mouth covered have inflamed mucus membrane. Consequently the natural conditions of the mouth, and of digestion, are interfered with, and as stated before, the question of how to restore the teeth and not interfere with the natural condition has baffled the skill and ingenuity of the dentists familiar with these facts. Wishing to do their patients no injury, if they can do them no good, they have refused to extract teeth firm in the jaw, and set their wits at work to resotre these diseased teeth instead of extracting them, knowing that they could never replace them.

Being familiar with these facts for many years, my efforts have been directed towards preserving the natural teeth and restoring what loss there might be without covering the roof of the mouth. My experience has convinced me that as a rule, a tooth firm in the jaw need not be extracted. There are but few exceptions. When the treatment is followed persistently, and proper judgment used, nearly all the partial loss of the teeth can be restored without covering the roof of the mouth, and made as valuable for masticating

No. 1.

food as the natural teeth, I am positive, and with less injury to the remaining teeth, than by any other method. The method referred to is that known as the "Low Method," or Bridge Work.

Bridge work consists in supplying vacancies between teeth or roots with artificial teeth, attached to the adjoining natural teeth or roots by means of bands or crowns, and held in such position that there is no contact with or pressure on the gums beneath, and thus no opportunity for secretion or other foreign matter to be held there and thereby become offensive.

There is really but one kind of bridge work, and but one way to make bridge work to insure success. There are many ways of making teeth without plate, but this is not bridge work. I will here try to explain in detail my manner of making and adjusting bridge work.

For the first illustration, as seen in cut No. 1, we have a case where all the teeth have been extracted, except the two cuspids and two second molar roots.

We first proceed to prepare the roots by crowning. I use gold crowns on the molar teeth and what is known as the Low Crown on the two cuspids.

The preparation of the two cuspids consists in making the crown ready for adjustment. I always measure the tooth to be crowned with gold, with a strip of block tin, 35 thick stub gauge or thereabouts. Place the tin around the tooth, and with plyers carefully measure the full size of the same.

Should you be measuring a tooth, or part of a tooth, on which there are projections, take the engine, and with a stone, grind off the same, making a smooth surface, so there will be nothing to interfere with the fitting of the bands properly. After cutting the tin measures by the marks made by the plyers you have the measures ready to make the gold bands by. Cut the bands and bevel the edges, and solder together and you are ready to fit. After fitting all the bands, and finishing the crowns in the usual way, I place each in position in the mouth, having previously regulated the articulation of each crown as desired, in the process of making. We now take a deep articulation in wax, and impression in plaster paris; remove before it gets too hard, and place all the crowns in their positions in the impression; varnish, oil and pour in the usual way; separate the cast from the impression, and place in the articulator. Then pour plaster. After the plaster has hardened, remove the wax and we have the articulation proper, and are ready to select and grind our teeth, having previously selected our shade. My experience has long ago taught me that no porcelain tooth can stand the pressure for bridge work. The strain on them being twice as great as with teeth on plates, which rest on the gums that give to pressure.

In order to prevent breakage of teeth and give strength, I have for many years been making a tooth with gold cusps. I will here describe my manner of doing so. I had some shells of bi-cuspids and molars made, or rather teeth, without the crown. They can now be found in some of the depots.

For the first step, I use 28 gauge platinum for a covering of the inside of the shell or just where you wish gold to flow. Then I bend the pins down to hold the platinum in position, and with a file, remove all over-lapping platinum to prevent breaking of our tooth in heating. The tooth is made flat on the crown surface with the express intention of restoring with a gold crown. This crown need not be very thick, but should perfectly resemble the cusps on the natural tooth, for the purpose of mastication. As these cusps are not on the market, and every dentist making bridge work cannot make it in a way to stand, without putting gold cusps on the grinding surface of the bi-cuspids and molars, I will here describe for the benefit of those who do not know how to make them, how they can be made with very little trouble. Pick out a natural tooth with cusps the exact shape you wish to have your gold cusps, mix some fire clay in a thick paste, then press your tooth into it a little deeper than you wish the cusps Having made the proper impression, remove the tooth, and set the impression over the gas stove to dry. After it is dried and reasonably hot, lay your pieces of gold in the impression and with a blow pipe, melt them. When melted, press with a piece of steel on the gold till cool. This mould will do to make many from. If you have not the fire clay and can get charcoal that is burned from fine grained wood and is soft, you can simply press your tooth into the charcoal and melt in the same way, or you can carve your teeth as you desire in a block of carbon. Of course, the little steel dies are handier, as we can swedge up our gold cusps in them, either solid or thin.

Having described our manner of making the cusps, we will now return to the manner of finishing our tooth. We left off by saying we covered the inside and bent down the pins and filed off the over-lapping platinum. We now place the cusp on the top of the tooth, and place in the position desired, holding it there with wax, and with a spatula trim the wax the exact shape we wish our tooth to be, v-shape, tapering from the crown down. We now encase in plaster and sand, which gives us a box. When hard remove the wax and place over the stove, and when sufficiently dry, fill in with coin gold, using the blow-pipe to melt it in a solid mass, and then our tooth is ready to file up and place in position on the articulator. Cut No. 2 shows the tooth in this condition.

No. 2.

After our teeth are all arranged, we hold the same in position with wax, remove from the articulator, encase with plaster and sand or asbestos in the usual way. That we may have a strong case, I always use platinum wire between each tooth, and then proceed to heat and solder. Be sure that all the gold cusps are are so arranged that you can get all soldered together, as this gives us great strength. My formula for solder, which I have

No. 3.

used for many years, and which will be found very easy flowing and almost the exact color of the gold you are using, is as follows: Always figure from the carat of gold you are working. Take one dwt. coin gold, 2 grains of copper, and 4 of silver. We now have our case soldered; after filing as desired, commence to finish with felt wheels and pumice stone, after which we use rough buff wheels. We are now ready to adjust in the mouth. In cut No. 3 we see the case ready for adjustment.

Have the assistant dry all the teeth or roots to be operated upon while you are mixing the cement. Be sure and use a kind which does not harden very rapidly or your cement will set before you get your teeth adjusted. Use sufficient cement to fill all the gold crowns perfectly when the case is driven to place. Moisten the step plugs and cap with cement, touching every portion, and with an instrument place a little cement in the bottom of the cavity. We now adjust our case, using the little rotor for the low crowns, and a piece of ivory for driving on the gold crowns. Cut no 4 represents the case when in position.

No. 4.

It will be seen by looking at the previous cut No. 3 that the teeth after having been soldered are all spaced fully one-third of the distance from the place of contact with the gums, and the grinding surface of the teeth, so that secretions could not possibly lodge there. I have given you a description of my manner of making a full upper case of bridge work, where there are roots to be crowned to support the bridge. I will now describe my manner of operating upon a case where the four centrals are missing, as seen in cut No. 5. To supply these four teeth where the cuspids are intact, I use a gold band.

I first measure the tooth with strips of tin, and make the gold bands, as before described, cut out the outside lower portion of the band before beginning to fit. In fitting as the band is being driven down, cut away any of the band that touches the gum before all touches; never drive the band under the gum, as inflammation would probably follow.

I mention this as I have seen many attempts to get rid of the band by driving up under the gums and cutting them out on the front, until they were too narrow for strength. It is hard work to make something out of nothing. The bands should be heavy and strong and the patient made to understand that if he expects to get rid of the annoyance of the plate he must sacrifice his dis-

No. 5.

like to showing gold. After driving the bands up close to the margin of the gums, as the cuspid teeth are very tapering, the bands will have to be taken in at the bottom. To do this I slit the band about a third of its length up, then place it on the tooth again, lap it over enough to bring it to a close fit, and then take it off and solder.

No. 6.

Continue taking it in wherever it does not perfectly fit the tooth, and after a good fit is obtained proceed as before described by taking an articulation and impression. In adjusting first try the case on to see that it fits and that the articulation is all right. Cut No. 6 shows the case ready for adjustment.

Next, have the assistant dry the teeth upon which the bands are going, and then mix your cement. This should be mixed to about the consistency of thick cream. It must be neither too thick nor too thin, or the adhesion will not be strong enough to hold. Cover your teeth with cement and then the inside of the bands. Place these on the teeth and carefully mallet up into position. For this purpose I use a steel instrument with a crease or groove in the end. The teeth must be kept dry after the case is in position until the cement is well set. After this is done bevel the edges of the bands and burnish close to the teeth, and if properly done they will be made to resemble gold fillings.

In cut No. 7 we have the case completed.

No. 7.

I am aware that in a case like this porcelain crowns instead of gold bands could be used, and I should consider it much preferable to do so where we have roots or unsound teeth to operate upon, but do not advise the destroying of nerves where the teeth are intact to supply such a case with crowns, as the bands will answer every purpose for many years.

If they should give out in after years the roots can then be crowned. I have many of these cases that have been in use seven and eight years, some of which have never loosened, and some I have re-set nearly every year. I always impress upon the patient the necessity of having them re-set immediately, should they become loose, and advise them to have their cases examined at least once a year. Should parties insist upon having crowns used to supply a case like the one just described on perfectly sound teeth, I should begin by using an aluminum disk, with corrundum, cutting deep as possible, both on the labial and lingual sides. Then use the excising forceps. This can be done under the influence of an anesthetic or otherwise. It is not by any means so painful an operation as one would think. If the nerve does not come out with the piece of tooth cut off, I take a piece of orange wood which I have previously cut the proper shape to drive into the nerve canal. I place it in creosote and let it soak a few minutes before beginning to operate. Immediately after severing the tooth, drive this into the canal, then remove, and dip in creosote and drive in again. This will perfectly fill the nerve canal; all sensitiveness will disappear, and you can begin to operate at once. I do not recommend this treatment for

No. 8.

sound teeth, but I have treated many exposed nerves in this way; also many teeth broken by accident, and think this the most satifactory way to dispose of such cases. I have never had any unfavorable results follow after operating upon teeth in this way, and I can hardly say as much in favor of any other treatment. I speak of this manner of treating exposed nerves as one of the operations that sometimes becomes necessary in adjusting a bridge properly. I do not claim any originality in this mode of treatment. I know several dentists who use this method, all of whom report satisfactory results. We now have cut No. 8 showing the roots prepared to receive the case.

I have many of these cases in use that are giving entire satisfaction. The instrument selected for preparing these roots should be one with small inside cutters and large bevelers, so as not to cut away any more tooth substance than possible.

No. 9.

Cut No. 9 represents the case ready for adjustment.

Cut No. 10 represents the case after adjustment.

No. 10.

In this article I have described my manner of making teeth for bridge work. I am now having made a tooth expressly for bridge work, which I hope to be able to place on the market soon. I have been using them, but have not perfected my shells and moulds sufficiently to enable me to get them out in large quantities.

The following cut No. 11 shows us a socket. These I propose to have ready made in various sizes in bi-cuspids and molars, with corresponding shells.

No. 11.

Cuts 12 and 13 represent the shells placed in sockets. Number 12 is a molar tooth showing the shell in position, and 13 is a central reversed.

No. 12.

No. 13.

Cut No. 14 represents the socket as made for the four central and two cuspid teeth. The advantage of these teeth can readily be seen not only for bridge work but all gold plates. A tooth, if broken, can readily be replaced without removing the bridge or cracking by soldering, and with only a small expense.

No. 14.

Cut No. 15 represents the shell placed in position in the socket which can be used for bridge or crown work, and will greatly reduce the labor in making either.

## Figures and Tables

**No. 1. f1:**
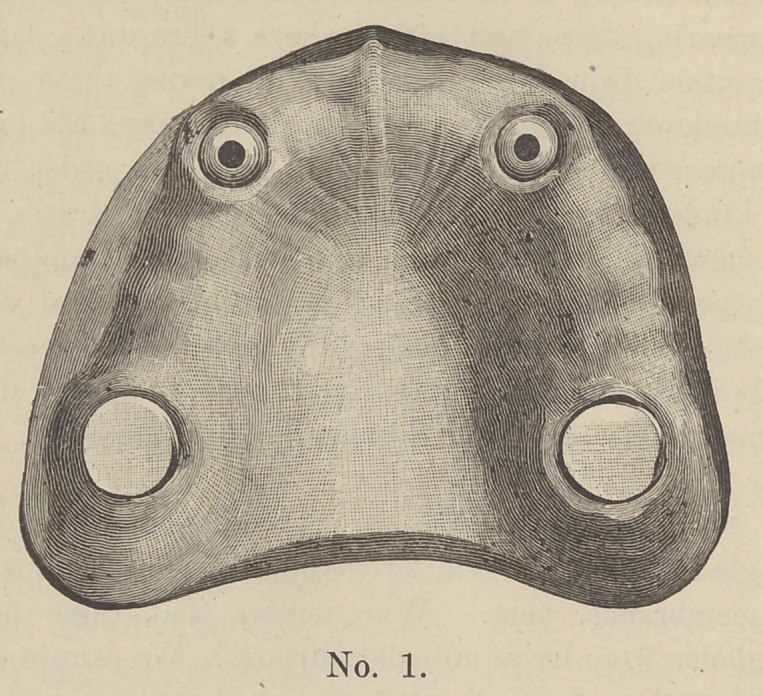


**No. 2. f2:**
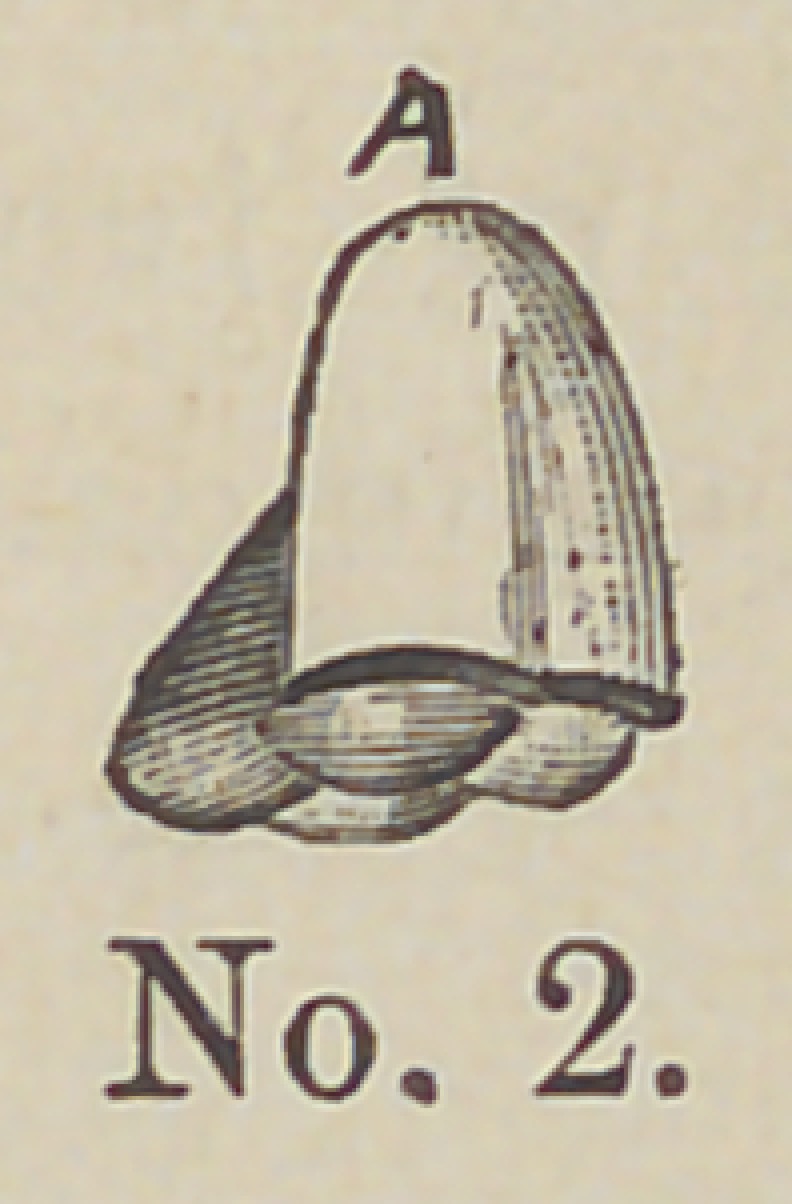


**No. 3. f3:**
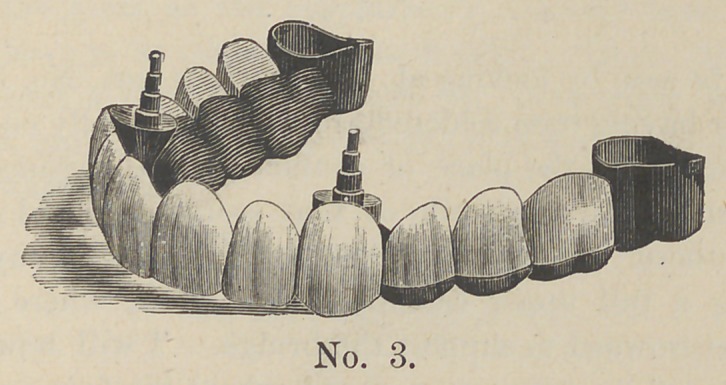


**No. 4. f4:**
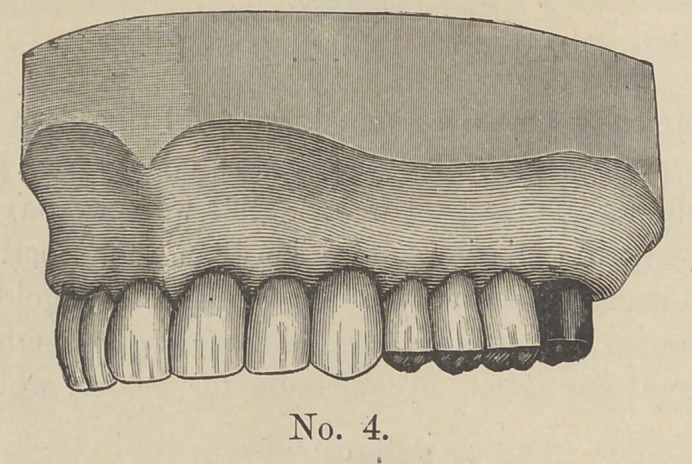


**No. 5. f5:**
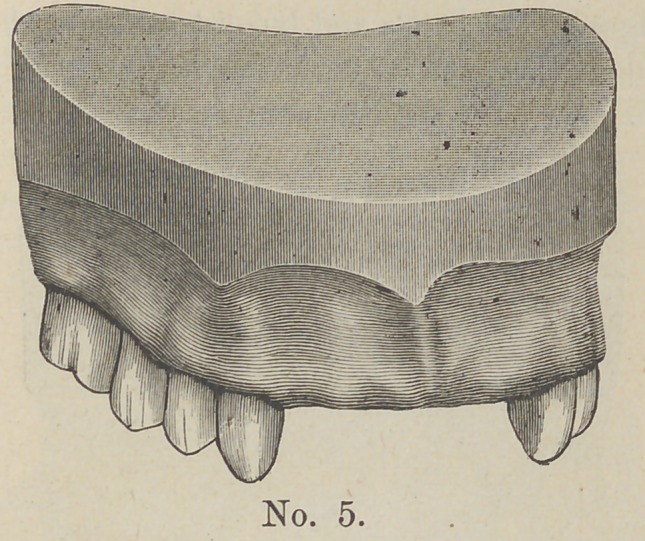


**No. 6. f6:**
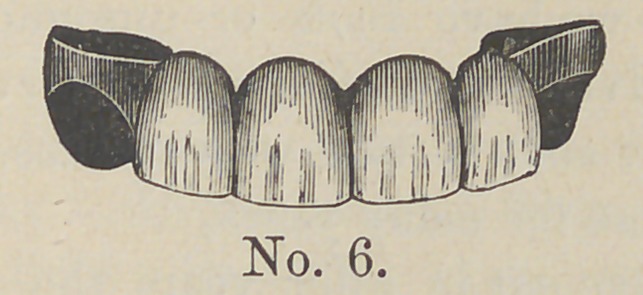


**No. 7. f7:**
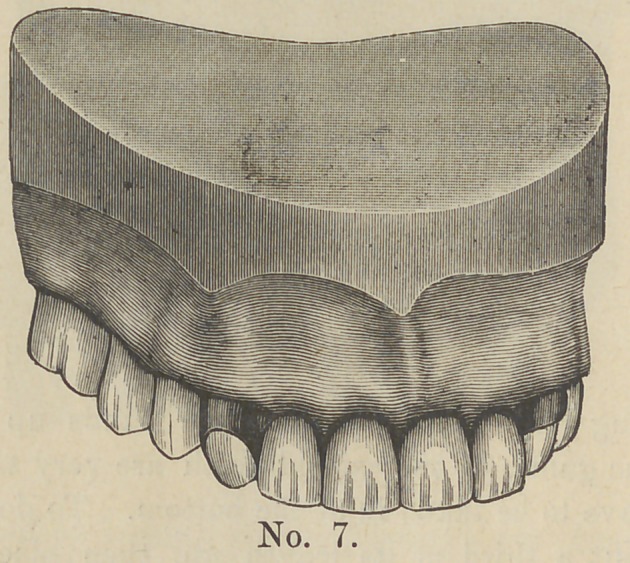


**No. 8. f8:**
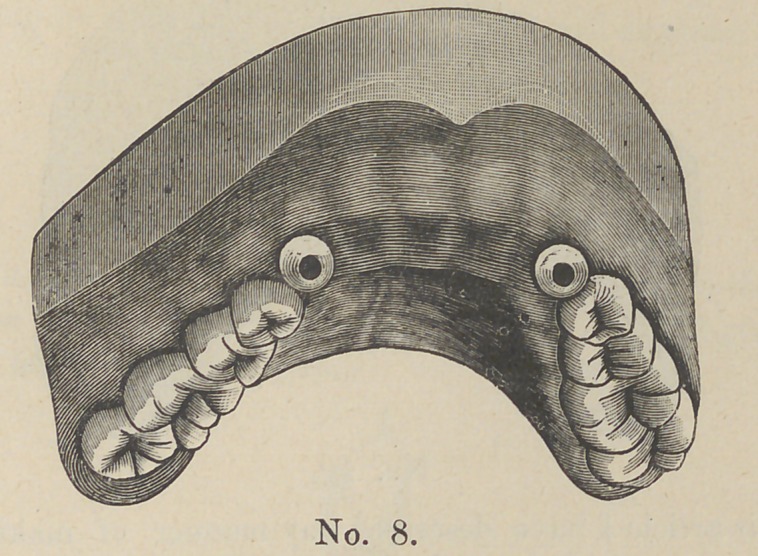


**No. 9. f9:**
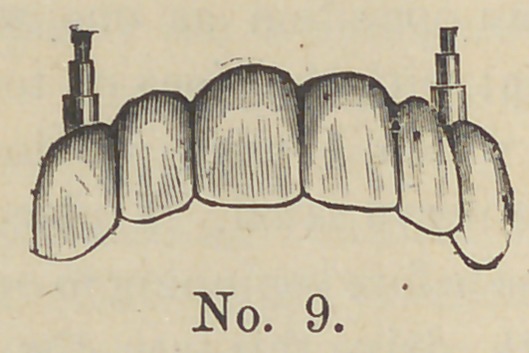


**No. 10. f10:**
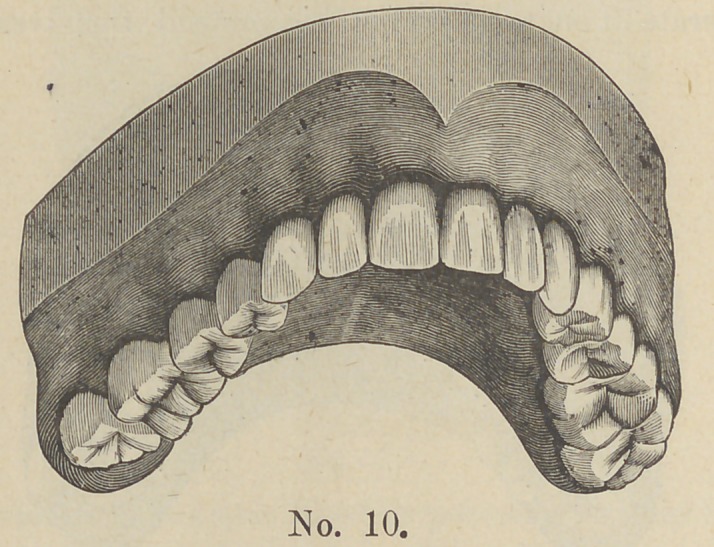


**No. 11. f11:**
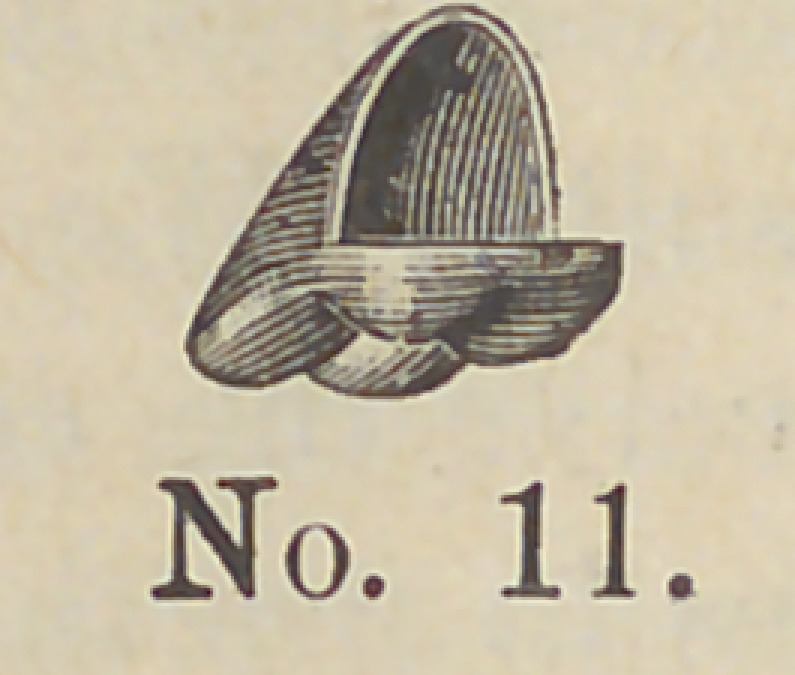


**No. 12. f12:**
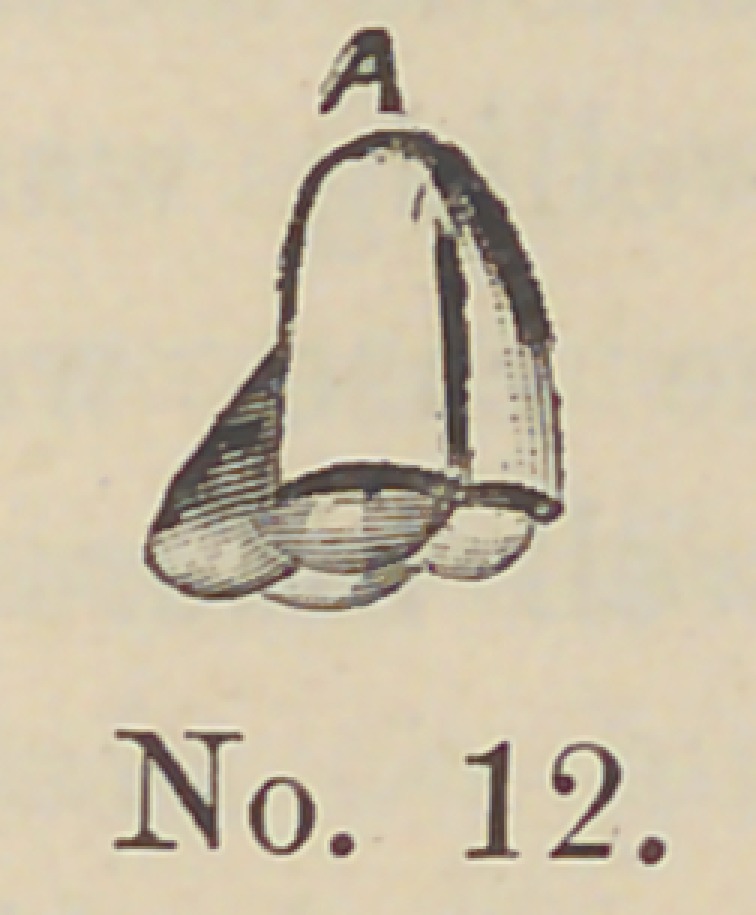


**No. 13. f13:**
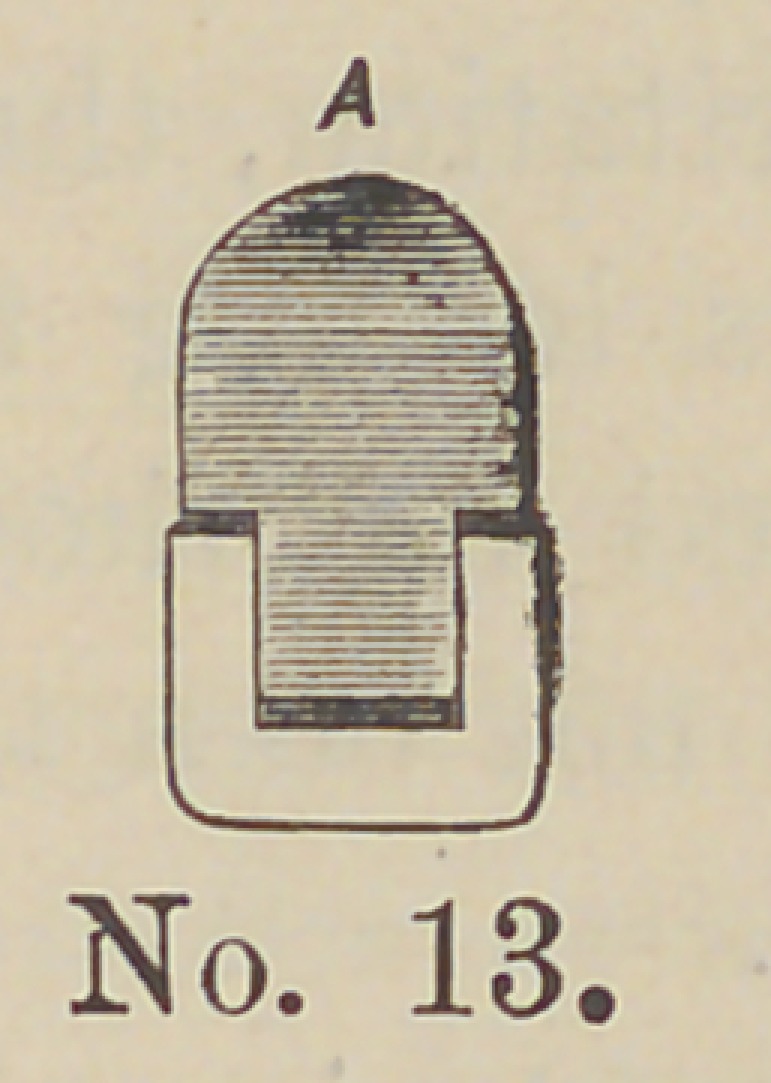


**No. 14. f14:**
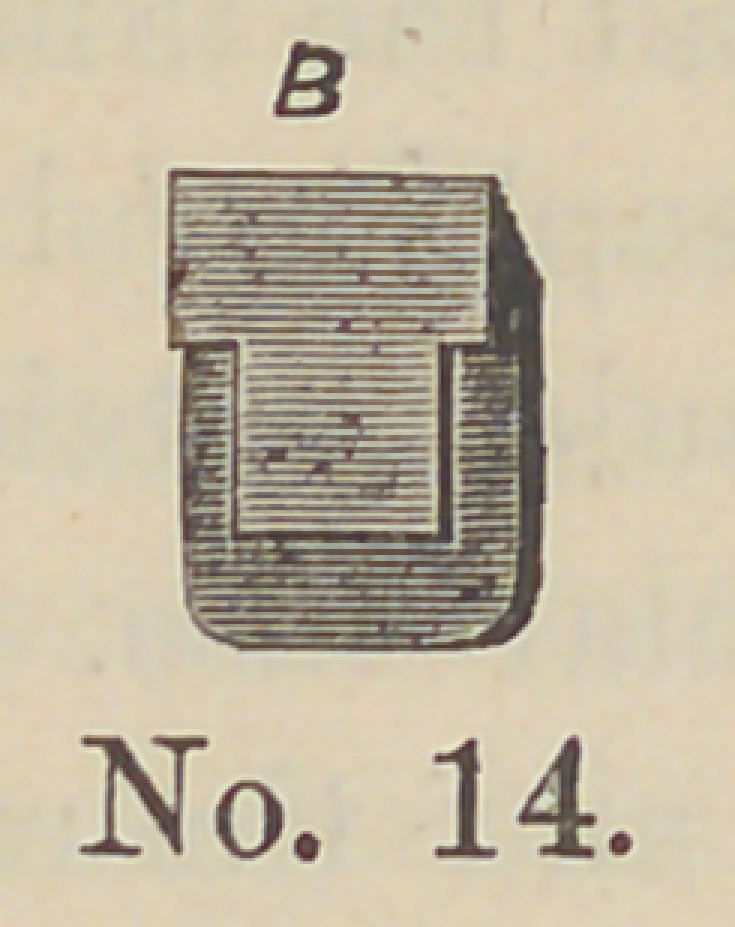


**No. 15. f15:**